# A qualitative analysis of dental professionals’ beliefs and concerns about providing aerosol generating procedures early in the COVID-19 pandemic

**DOI:** 10.1038/s41405-022-00094-9

**Published:** 2022-01-14

**Authors:** Matthew Cousins, Kajal Patel, Mariana Araujo, Laura Beaton, Claire Scott, Douglas Stirling, Linda Young, Jennifer Knights

**Affiliations:** 1 NHS Tayside, Dundee, UK; 2 NHS Education for Scotland, Edinburgh, UK

**Keywords:** Guidelines and law in dentistry, Infection control in dentistry

## Abstract

**Introduction:**

In response to the COVID-19 pandemic, the Scottish Dental Clinical Effectiveness Programme (SDCEP) initiated a rapid review of the evidence related to the generation and mitigation of aerosols in dental practice. To support this review, a survey was distributed to better understand the provision of aerosol generating procedures (AGPs) in dentistry.

**Methods:**

An online questionnaire was distributed to dental professionals asking about their current practice and beliefs about AGPs. Data were analysed using qualitative content analysis.

**Results:**

Analysis revealed confusion and uncertainty regarding mitigation of AGPs. There was also frustration and scepticism over the risk of SARS-COV-2 transmission within dental settings, the evidence underpinning the restrictions and the leadership and guidance being provided, as well as concern over financial implications and patient and staff safety.

**Discussion:**

The frustration and concerns expressed by respondents mirrored findings from other recent studies and suggest there is a need for reflection within the profession so that lessons can be learned to better support staff and patients.

**Conclusion:**

Understanding the profession’s views about AGP provision contributed to the SDCEP rapid review and provides insights to help inform policymakers and leaders in anticipation not only of future pandemics but in considering the success of any large scale and/or rapid organisational change.

## Introduction

On 23 March 2020, the United Kingdom (UK) entered a state of national lockdown due to the ongoing COVID-19 pandemic. Directed by the four UK Chief Dental Officers (CDOs), most non-urgent dentistry ceased [1]. Dental practices were initially advised to triage patients by telephone and provide either advice, analgesia, or antibiotics as appropriate. In all four UK nations arrangements for the provision of urgent dental care were put in place. In Scotland the Scottish Dental Clinical Effectiveness Programme (SDCEP) responded to provide resources to support practices in these early days, adapting existing guidance in relation to the management of acute dental problems [2].

Of particular concern in dentistry was the ramifications of the pandemic for the provision of aerosol generating procedures (AGPs) [3]. To inform future policy and the development of guidance for the re-instatement of AGPs within general dental practice, in July 2020 National Services Scotland released a rapid literature review of the available evidence, providing ‘a critical appraisal of the fundamental requirements for ventilation as a control strategy along with other mitigating requirements and the modelling of AGPs with reference to the site and surrounding area’ [4]. The review stated that a ‘fallow time’ was required following an AGP to allow the aerosols produced to be dispersed. Definitions were provided for air changes per hour (ACH), the difference between natural and mechanical ventilation, and details set out on the impact mitigations, such as rubber dam, high-volume suction, pre-procedural rinsing, ventilation and air clearance systems, would have on fallow time. Recommendations on infection control measures were described as having to take account of the ‘balance of estimated risk with consideration of feasibility and appropriateness’ at a time where decisions were necessarily having to be made on the basis of limited evidence.

On 7 August 2020, a CDO Scotland letter advised that some dental practices would be able to ‘opt-in’ to providing a limited range of AGPs to registered NHS patients in need of urgent care from 17 August [3]. This would be permitted if the practice ‘replicated the arrangements in UDCCs [Urgent Dental Care Centres], including enhanced PPE [personal protective equipment]’. For surgeries where ACH ≥10 could be evidenced, a minimum 20 min fallow time was set. For ACH <10, or where there was no data available, the minimum fallow time was 60 min and if there was no external ventilation (natural or mechanical), then no AGPs were permitted. At this time not all dental practices were expected to be ready to provide AGPs [3].

In anticipation of the reintroduction of AGPs in general dental practice, SDCEP initiated a rapid review of the evidence related to the mitigation of AGPs in dentistry and the associated risks of SARS-CoV-2 transmission. It aimed to reach agreed position statements, based on an appraisal of the available evidence and other relevant factors, to inform policy and clinical guidance. To support this review, a questionnaire was distributed to dental professionals to determine the current provision of AGPs and capture practitioners’ beliefs and concerns. The results were presented to the SDCEP rapid review working group, to inform the SDCEP rapid review *Mitigation of Aerosol Generating Procedures in Dentistry*, which was published in September 2020 [5]. This paper reports findings from the qualitative data obtained. Quantitative findings will be reported separately.

## Methods

### Sample and recruitment

Dentists, therapists, hygienists, and hygienist–therapists in the General Dental Service (GDS), Public/Community Dental Service (PDS/CDS) and Hospital Dental Service (HDS) were invited to complete the questionnaire. It was disseminated to dental professionals registered in Scotland with an NHS Education for Scotland (NES) Portal account and who had opted in to receive marketing communications. NES Portal accounts are limited to practitioners in Scotland. Therefore, those recruited from outside of Scotland or those without NES Portal accounts were introduced to the questionnaire via Twitter, and professional networks known to the SDCEP AGP Review working group.

### Data collection

The anonymous online questionnaire was split into three sections collecting quantitative and qualitative data on: respondents’ current provision of AGPs, mitigation factors used and beliefs about minimising the risk of SARS-COV-2. Qualitative data was gathered using two free-text boxes at the end of the ‘mitigation factors’ and ‘beliefs about minimising the risk of SARS-COV-2’ sections. Demographics collected included country of residence, usual work setting and professional group. The questionnaire was hosted on Questback, an online survey tool used by NES, and was open from 18 to 24 September 2020.

### Data analysis

The qualitative responses were analysed using qualitative content analysis, a method for analysing written, verbal, or visual communication messages, which involves identifying and coding key categories within the data [6, 7]. The data was coded and analysed by MC and KP. If there was a disagreement with regards to the coding or analysis, this was discussed with senior colleagues (MA, LB, JK, CS) until a consensus was reached.

## Results

Respondents were asked to expand on their responses to questions regarding mitigation currently in place in their practice and their personal views around AGPs and the risk of SARS-COV-2 transmission. Out of a total of 2847 questionnaire responses, 603 (21%) respondents provided a comment for at least one of the two free-text questions; there were 318 (11%) comments for the question regarding mitigation currently in place in their practice and 433 (15%) comments for the question regarding their personal views around AGPs and the risk of SARS-COV-2 transmission. Demographic results for the 603 respondents who provided a comment for at least one of the two free-text questions are presented in Table 1.

**Table 1:** Demographics.

Professional role	Dentist	90%
	Therapist/hygienist/hygienist therapist	6%
	Dental nurses	2%
	Practice managers	1%
	Other	1%
Work setting	GDS	86%
	PDS/CDS	8%
	HDS	2%
	Private practice	2%
	Other	2%
Country of work	Scotland	40%
	England	47%
	Wales	9%
	Northern Ireland	3%
	Other	1%

Qualitative content analysis revealed eight main themes relating to the beliefs and concerns of dental professionals regarding the provision of AGPs at the time of data collection.

### Mitigation of AGPs

Respondents shared their uncertainty about the correct use and effectiveness of mitigating factors and there was variation in the methods being employed; when discussing the environmental mitigating factors of natural and mechanical ventilation, various challenges were described for example, non-opening windows and the effects of adverse weather. Several respondents reported being unable to accurately calculate ACH, particularly when using natural ventilation. Other challenges included meeting the financial costs associated with installing mechanical ventilation devices and a lack of trust in external suppliers. Guidance on how to calculate ACH was described by one respondent as ‘inadequate’.

‘I feel the NHS SOP [Standard Operating Procedure] regarding ACH is both poorly explained and rests on very poorly understood scientific basis of theoretical risks… There has also been little to absent guidance on how to quantify air change’ (GDS Dentist)

Mixed responses were received regarding air scrubbers/cleaners; the majority suggesting a lack of practitioner knowledge about these devices and concern around how these were not yet included in the NHS SOP.

Several types of procedural mitigation were discussed. The use of rubber dam received mixed responses with regards to its effectiveness and ease of use. Whilst some emphasised the belief that aerosol transmission would be reduced by using rubber dam, others expressed concern over time constraints and patient tolerability. Comments regarding pre-procedural mouthwashes were predominantly critical, with concerns raised regarding their use with paediatric patients. High-volume aspiration use was predominantly met with positivity.

### Fallow time and cleaning

There was a significant amount of uncertainty expressed regarding what constituted the end point of an AGP and concerns about the consequences of this uncertainty. Some respondents described booking appointment lengths with the ‘worst-case scenario’ in mind (i.e. finishing the AGP at the end of an appointment). Correspondingly, there was uncertainty and differing beliefs about what constituted the start point for fallow time, with respondents suggesting that fallow time should start: from the moment the AGP was finished when the high-speed handpiece was put down; when everyone had left the room; at the end of treatment or appointment time; when one stopped using the ultrasonic scaler or airflow; when the patient put their mask on before leaving; following removal of barrier wrapping; or after the last use of the 3-in-1 triple syringe. Many respondents also raised concern about the additional time required for preparation prior to appointments and for cleaning afterwards.

‘Whilst fallow time is a major component, the cleaning time plus drying of the floor following mopping increases the time between procedures to by at least 50% of the fallow time. This should not be forgotten’ (GDS Dentist)

There was variation in descriptions of when respondents believed cleaning times should commence: as soon as the appointment finished; 45 minutes into the fallow time; 10 minutes after fallow time commenced; at least 20 minutes after fallow time starts.

The increase in fallow time had led to a significant reduction of capacity in dental practices as well as administrative difficulties in scheduling appointments so that patients in different surgeries did not cross paths. Some dental professionals explained how they prioritised AGPs in terms of different categories and time requirements (e.g. low and high risk, short AGP) or used an alternative dental material for restorations in certain instances to prevent further delay of appointments.

Questions were raised about who should be determining fallow time for dental practices. The CDO for Scotland’s letter regarding remobilisation advised dental practices to liaise with their local Health Boards around this matter. However, some respondents commented that regardless of the mitigation processes implemented, 1 hour remained the local advice and this may have been acting to discourage practices from investing in other measures, such as mitigation devices.

‘Seems pointless to investigate means of reducing fallow time if health board won’t allow for AGP fallow time to be reduced from the 1 hour restriction’ (GDS Dentist)

### Evidence and guidance related to mitigation of aerosols within the dental setting

Respondents described their uncertainty and confusion around what was and was not appropriate practice in terms of AGPs and mitigation. There was an urgent call for clear and nationally agreed guidelines.

‘I feel I’m going around in circles looking for the best advice to be sure that at some point in the future, I can prove what we are doing is adequate at the least. In the meantime, we seem to be on our own deciding what is best & appropriate’ (GDS Dentist)

Serious concerns were raised by several respondents around the sufficiency of the evidence base upon which guidance was being developed.

‘We need hard evidence of what we are being told is necessary. There is, at present, a mixed bag of confusion out there’ (GDS Dentist)

Having too many sources of information and advice, for instance from bodies such as the General Dental Council (GDC), the Faculty of General Dental Practice (FGDP), the CDOs, the Health and Safety Executive, Public Health England (PHE) and defence societies, was described as causing difficulties for senior staff and dental professionals when deciding upon protocols and formulating risk assessments and SOPs for their practice.

‘The chief problem has been that there are so many sources of guidance and that they conflict with one another on important matters, such as fallow periods’ (GDS Dentist)

Respondents described making use of social media, reviewing professional dental forums, attending webinars and online tutorials and talking to other dentists to try and make sense of the situation and take appropriate steps in the absence of advice from any one central/single body.

### Risk of transmission of SARS-CoV2 in dentistry

Frustration was expressed over what was felt to be over-regulation of the profession and prevention of dental professionals being supported to exercise their clinical judgement.

‘We need to see our patients for their benefit but these rules mean that although we are competent and professionals we aren’t able to work to see and treat them… You won’t find a more clinical, clean and well managed profession like dentistry so let us do what we are trained…to do’ (GDS Dentist)

Risk of transmission in the dental setting was described by some respondents as already being minimal due to screening processes and pre-existing cross-infection control measures.

Some also queried the extent to which coronavirus was present in dental practices during the months of January to March 2020, when extra preventative measures were *not* being taken:

‘Surely if aerosol transmission was an issue then clusters related to dental practices would have been identified in February/March and internationally?’ (GDS Dentist)

One respondent made the argument that previous infectious diseases had not affected dental practices disproportionately to other sectors, nor were precautionary measures taken to the same extent.

‘There is very little evidence of Hepatitis B, Hepatitis C, Influenza or HIV being transmitted by Dentists to their patients. The same applies to SARS, MERS, Swine Flu and Avian flu and during those infections dentistry in the UK did not alter’ (GDS Dentist)

The sense that dentistry was being unjustly treated in the context of the COVID-19 pandemic was also found in responses which made comparisons with the experiences not only of other clinical professions such as medicine, but also with other sectors such as the hospitality industry and the mitigation steps being required of restaurants and bars. Some respondents expressed frustration that medical and dental aerosols were being considered as similar entities, judged to have the same risk of transmission of COVID-19 and therefore requiring similar levels of mitigation and PPE.

‘I do think comparing dental aerosol (which is vast majority from dental lines and in our case is antimicrobial) with aerosol from intubation (100% from patient) is ludicrous!’ (GDS Dentist)

### Implications of the pandemic on patient care

Several respondents expressed concern about the impact of reduced practice capacity on patient care.

‘Access to NHS dental services is severely compromised as a result of the 60 min fallow time and this is causing major issues with access to dental care and is exacerbating oral health inequality’ (GDS Dentist)

Anxiety was expressed regarding patients coming to harm because of dentists not being able to carry out routine fillings or undertake screening for malignant diseases due to reduced patient throughput.

‘I really hope fallow time can be reduced as patients and practices are suffering. We have a huge backlog of patients to see. Just today I carried out restorative work on two patients that had a small amount of caries in Feb/March and today they were huge cavities!’ (Hygienist Therapist)

One respondent felt that this compromised care was a result of government failings.

‘The patients in my practice have not received the care they ought to have due to incompetence by Scottish Government to deal with this properly. People have been over-prescribed antibiotics and endured pain’ (GDS Dentist)

Concern was raised about possible growing frustration amongst patients due to being unable to access definitive treatment and a lack of patient understanding regarding restrictions such as fallow time.

### Financial implications for patients and dental professionals

Respondents voiced concerns regarding the implementation of fallow time and necessary reduction in patient throughput, viewing this as a major prohibitor for financial turnover. Some warned of the very real possibility that dental practices could close.

‘I cannot see how self-employed associates can earn a living with 1 hour fallow time never mind a practice stay afloat through NHS dental provision in the guise of SDR [Statement of Dental Remuneration] pre-COVID. The business model is fundamentally flawed with the necessity for volume’ (GDS Dentist)

Some respondents believed that the different funding models between NHS and private practice were introducing inequities; they felt uncomfortable offering some treatments privately when they were unable to provide them as NHS treatment. A small number of respondents noted a divide between NHS and private dentistry regarding the roles of dental professionals; private practices were still operational but staff working in NHS practices were often furloughed.

‘Ridiculous how NHS practices are getting so much money with their staff on furlough pretending they aren’t allowed to treat patients’ (GDS Dentist)

Others suggested that there had been little or no financial support for the whole profession during this period. The potential for redundancies was also highlighted as a consequence of the restrictions imposed and/or the ceasing of government financial support schemes. Costs associated with making practices safe were also highlighted as an additional financial burden.

‘I have no idea where to begin looking for mechanical ventilation and those that I have seen in dental specific sectors are extortionate. I have already taken out another loan to keep my practice going through COVID-19 and I cannot afford further investment’ (GDS Dentist)

### Mental and physical impact on members of the dental team

Several respondents highlighted the toll that the pandemic was taking both personally and professionally on dental professionals. Concerns were raised around staff health and safety and the challenges of working under the current levels of PPE.

‘It is very stressful to work in the full PPE and adds considerable stress to an already stressful job’ (GDS Dentist)

‘Undertaking AGPs with the current level of PPE is extremely challenging for the staff and may theoretically reduce risk of transmission but poses other threats in terms of health and wellbeing of the staff’ (GDS Dentist)

A specific issue regarding out-of-date PPE was raised.

‘The masks provided are sometimes a few years out of date. It is unclear how these have been recertified by the NHS for the manufacturer states this should not be done… It is difficult to reconcile how an unknown recertification method complies with the relevant ISO standard’ (HDS Dentist)

Issues raised were not limited to physical safety, the emotional wellbeing of dental professionals was also a concern with one GDS Dentist suggesting that ‘*the mental health of dentists has suffered terribly.*’

Concerns about wellbeing were also evident in relation to other members of the dental team.

‘My nurses are upset having to deal with other people’s problems on the phone and at the front door, one nurse was really upset on Monday and won’t answer the phone now’ (GDS Dentist)

Hygienists and therapists reported feeling ‘*woefully undervalued’* and having lost paid working hours and surgery space due to the need for surgeries to fulfil the fallow time requirements.

‘Hygienists and therapists are very much high risk, but expected to do non-AGP which is proving extremely difficult physically, correct equipment is rare to carry out this job. Its causing a lot of stress amongst the profession, with many leaving jobs to take a stance due to poor conditions and impossible expectations set by principals’ (Therapist)

Concern was also raised around the possibility of experienced dental nurses in the GDS leaving the profession due to discontent with remuneration in comparison to nursing colleagues in hospital settings. Job dissatisfaction amongst some dentists was also evident.

‘The job has lost all its fun and I would pack in now if I could’ (GDS Dentist)

‘I also fear that the profession will lose experienced caring professionals who struggle to cope with the additional burden’ (GDS Dentist)

### Dental professionals’ opinions on the leadership shown throughout the pandemic

Some respondents expressed frustration and disaffection with leaders in the profession, citing mixed messages, lack of direction and unnecessary delay in the provision of coherent guidance. It was also suggested that these perceived leadership failings were causing divisions within the profession and could adversely affect patients.

Discontent was also directed at professional bodies and other organisations (e.g. PHE, Public Health Wales, GDC) and there was concern that some dental professionals felt left to undertake their own research or rely on colleagues’ help.

‘It has taken great effort to find this advice. The profession[al] bodies have been pathetic in leading’ (GDS Dentist)

While some felt that there was a damaging disconnect between the leaders in the profession and dental staff ‘at the coal face’, others reserved criticism on the basis of unprecedented circumstances.

‘From what I’ve read/heard nobody knows the best way to manage things so we are all in the dark to an extent’ (GDS Dentist)

Concerns were also reported around communications and the provision of guidance and support at a local level. A SOP from one Scottish Health Board was described as ‘*appalling*’ and containing ‘*nothing helpful at all.*’ Another response suggested that current practice in England was for individual NHS Trusts to interpret guidance extrapolated from other medical procedures. A lack of understanding in local Health Boards of dentistry in terms of procedures and risks was also raised in relation to fallow time and the organisation of assessments.

‘As I am an employee the Trust has organised all assessments however this also worries me as they are slow to act and are too remote from dentistry to understand the serious risks within our work’ (HDS Dentist)

‘I asked my local health board for help in determining if and how we might be assessed for our fallow time reduction—and was told that this was too complicated for them to assess each Practice, as they didn’t have the appropriate training/skills’ (GDS Dentist)

## Discussion

The results of this survey provide a snapshot of the beliefs and concerns of dental practitioners during the short time period between the re-instatement of AGPs and the publication of the SDCEP rapid review, and, subsequently, the COVID-19 infection prevention and control dental appendix produced by PHE [8]. The results show considerable uncertainty within the profession around what mitigations should be used, their individual and collective benefit, how to calculate ACH, and when fallow time should commence. This is not especially surprising considering that the data was collected in the period between the re-instatement of AGPs yet prior to the release of the SDCEP rapid review. A critical juncture for dentistry in the UK, the decisions taken by leaders in the profession and other key actors at this time would inform the direction of both the clinical practice and the financial future of many of those working in general dental practice.

The COVID-19 pandemic may be the first time that many healthcare professionals have had to deal with a long-term, unpredictable and seemingly uncontrollable situation [9]. There was a sudden shift from a position of secure clinical knowledge and experience, with relative control over the work environment, to a situation of high uncertainty where no one ‘right answer’ could be readily supplied. This might be experienced by clinicians as unsettling, and contrary to the notion embedded in much learning curricula and professional culture that clinical certainty, or at least agreed and unified solutions, can be reached [10, 11].

Feelings of frustration and scepticism were evident, particularly the perceived lack of robust evidence about the risk of transmission of SARS-CoV-2 in a dental setting and how therefore evidence was being used to inform recommendations made by dental regulatory bodies. Indeed, at the time of data collection, there *was* a great deal of uncertainty regarding risk of transmission in dentistry, with the available evidence being constantly updated as new findings were published [12–14]. In light of this uncertainty, and amid calls for clearer evidence, SDCEP’s rapid review appraised the available evidence related to AGPs and reached agreed positions that could be used to inform policy and clinical guidance [6].

In the face of this uncertainty, and before the SDCEP rapid review was published, dental professionals sought advice from a range of sources, including professional bodies (e.g. FGDP, British Dental Association), government, and local Health Boards. As Coulthard notes, many dental professionals ‘looked to the NHS for national guidance’ but found ‘discrepancies’ between the four UK nations and, initially, advice that was not aligned to the emerging evidence from countries who were ahead of the UK with regard to COVID-19 transmission [15]. Among the respondents, this situation resulted in increased frustration and confusion, with several respondents criticising leadership at individual, organisational and government levels. However, as noted above, this was an unprecedented time for dentistry, and healthcare more generally, with healthcare professionals and leaders at all levels having to quickly adapt to new ways of working and grapple with the unknown. Crisis leadership has been previously described as one of the most important yet understudied factors in crisis management and while planning is important, leadership, particularly in the aftermath of a crisis, may ‘trump any preparation’ [16]. Now would seem a critical time to reflect upon, investigate and analyse what has taken place over the course of the last two years so that lessons can be learned from this crisis to strengthen leadership and better support staff and patients [17, 18].

Significant concern was expressed about both the financial implications for dental practices and job security. The mental health and wellbeing of both staff and patients was also highlighted. In a cross-sectional study of dentists and dental health professionals in primary dental care and those in training, undertaken between June and October 2020, 27% of respondents reported significant depressive symptomology (compared with 18% in a population-based cohort in normal conditions) and 55% of primary care staff rated themselves as emotionally exhausted [19]. The same study also found that over half of primary care staff felt unprepared financially for the effects of the pandemic and concerns were also raised about the impact on the oral health of patients. These findings resonate with various concerns about the oral health of patients expressed by respondents to this survey. The wellbeing of staff, as well as patients, must be at the forefront of decision-making around the imposition, or lifting, of restrictions related to the provision of dental care.

Given that the survey responses were received from a sample of the profession at a very specific stage of the evolving response to the pandemic, the results should not be assumed to be representative of the whole professional population nor considered to be representative of the beliefs and concerns amongst the profession as the pandemic further progressed. The research team acknowledge the effect of researcher position and to ensure credibility maintained a reflexive approach through regular team discussion and deliberation. The collection of both quantitative and qualitative data sets in the online questionnaire had the benefit of providing enhanced explanations of a complex situation.

The results of this study suggest that now is a crucial time for stakeholders and regulatory bodies, with input from various levels within the profession, to reflect on the future of NHS dentistry in the UK, around, for instance, how to create a system that is more resilient, responsive and equitable and better prepared for current and future challenges [20, 21]. The results from our study demonstrate discontent, confusion, fear, anger, and distrust amongst the workforce. These emotional responses, along with concerns about mental wellbeing, ought to be carefully considered moving forward.

## Conclusion

Understanding the profession’s views about AGP provision was an important factor in the development of the SDCEP rapid review of the evidence related to the mitigation of AGPs in dentistry and the associated risks of SARS-CoV-2 transmission. Responses to the questionnaire designed to support the review revealed how dental professionals felt about AGPs, mitigation factors and methods of minimising the risk of SARS-COV-2 transmission at the time of data collection.

There was a great deal of uncertainty and associated concern regarding provision of dental care and the impact of restrictions on patients and practitioners. This paper will be of particular interest to policymakers and leaders in the field of dentistry, or anyone involved in the strategic or operational planning of dental services, in anticipation not only of future pandemics but in considering the success of any large scale and/or rapid organisational change.
